# Sequential treatments with TRK inhibitors in a patient with *NTRK* fusion-positive sarcoma: A case report

**DOI:** 10.1097/MD.0000000000036232

**Published:** 2023-12-08

**Authors:** Yuta Kubota, Masanori Kawano, Tatsuya Iwasaki, Ichiro Itonaga, Hiroshi Tsumura, Nobuhiro Kaku, Kazuhiro Tanaka

**Affiliations:** a Department of Orthopaedic Surgery, Oita University Faculty of Medicine, Yufu City, Oita, Japan; b Department of Advanced Medical Sciences, Oita University Faculty of Medicine, Yufu City, Oita, Japan.

**Keywords:** entrectinib, larotrectinib, *NTRK* fusion-positive sarcoma, TRK inhibitor

## Abstract

**Rationale:**

Precision medicine and tumor-agnostic treatment strategies have recently been promoted for clinical use. One of the most successful treatments in patients with neurotrophic tyrosine receptor kinase (*NTRK*) fusion-positive tumors is targeting the tropomyosin receptor kinase (TRK) with an inhibitor. The TRK inhibitors, larotrectinib, and entrectinib, have been approved in many countries. Nevertheless, the most effective administration regimen for these TRK inhibitors is uncertain. To date, no reports have shown the efficacy of sequential treatment with larotrectinib and entrectinib in patients with *NTRK* fusion-positive tumors. In this report, we present a patient with *NTRK* fusion-positive sarcoma arising from the anterior mediastinum, with tumor progression after 4 months of entrectinib use. The patient took larotrectinib subsequently and maintained disease control for more than 21 months.

**Patient concerns:**

A 48-year-old female visited a physician because she experienced difficulty in breathing and chest and back pain with no obvious cause 2 months ago. Computed tomography (CT)-guided biopsy was performed at a district general hospital, and histopathological examination revealed a small round cell tumor. She was referred to our hospital, and a second CT-guided biopsy was performed to confirm the pathological diagnosis. Considering the results of the histopathological examination, Ewing sarcoma was suspected, but a specific fusion gene was not detected due to poor quality specimens.

**Diagnoses:**

After 3 regimens of cytotoxic chemotherapy, biopsy was repeated, and specimens were analyzed using next-generation sequencing. The *PHF20-NTRK1* fusion gene was detected, and the tumor was finally diagnosed as an *NTRK* fusion-positive sarcoma.

**Interventions:**

She was administered the TRK inhibitor entrectinib, but the tumor started to grow after 4 months of medication, and she stopped taking entrectinib. After 1 cycle of cytotoxic chemotherapy, another TRK inhibitor, larotrectinib, was administered.

**Outcomes:**

Her stable disease was maintained for more than 21 months. Here, we have shown that sequential administration of both drugs can be effective.

**Lessons:**

In the treatment of *NTRK* fusion-positive tumors, there are cases in which 2 approved first-generation TRK inhibitors can be used sequentially.

## 1. Introduction

Recently, owing to next-generation sequencing, gene panel tests and molecular targeted drugs have been introduced, along with the promotion of precision medicine and tumor-agnostic treatment strategies. One of the most successful treatments for patients with neurotrophic tyrosine receptor kinase (*NTRK*) fusion-positive tumors is tropomyosin receptor kinase (TRK) inhibitor treatment. The TRK inhibitors, larotrectinib and entrectinib, were approved by the United States Food and Drug Administration as a selective *NTRK* inhibitor and multi-kinase inhibitor in 2018 and 2019, respectively. Approval was also obtained in Europe and Japan. In Japan, entrectinib and larotrectinib were approved in February 2020 and March 2021, respectively. From the outset, it was assumed that using first-generation TRK inhibitors, such as larotrectinib or entrectinib, would render *NTRK* fusion-positive tumors resistant to such drugs. Therefore, next-generation TRK inhibitors, such as selitrectinib and repotrectinib, have been developed and planned for clinical trials along with first-generation TRK inhibitors. However, the optimal way to utilize these TRK inhibitors remains uncertain. To date, no reports have shown the efficacy of sequential treatment with larotrectinib and entrectinib in patients with *NTRK* fusion-positive tumors.

Here, we present a patient with *NTRK* fusion-positive sarcoma arising from the anterior mediastinum, with tumor progression after 4 months of entrectinib administration. Subsequently, she took larotrectinib and maintained disease control for more than 21 months.

## 2. Case report

A 48-year-old female visited a physician because she had experienced difficulty in breathing and chest and back pain with no obvious cause 2 months ago and developed fever, coughing, and hoarseness. Her medical history included mastectomy for breast cancer aged 40 years and cholecystectomy. The patient family had no relevant medical history. She had migraines and was allergic to eggs and buckwheat. As a mediastinal tumor was suspected on chest radiography, she was referred to a district general hospital. Computed tomography (CT)-guided biopsy was performed at the hospital, and histopathological examination revealed small round cell tumors, suggesting small cell carcinoma or Ewing sarcoma. Therefore, she was referred to our hospital for a detailed diagnosis and treatment.

Upon physical examination, she was able to walk with exertional breathlessness. Additionally, she had a hoarse voice and dry cough. Her body temperature was 38 °C, and she felt pain in the neck, spreading to the bilateral shoulders. On auscultation, her bilateral breath sounds were weakened (R > L), but no rattle was heard.

Laboratory tests revealed no remarkable abnormal data, including in blood count and blood chemistry test results. Tumor marker tests such as serum carbohydrate antigen 19-9 were within the normal range, except for a serum squamous cell carcinoma antigen level of 1.8 ng/mL (reference range, ≦1.5 ng/mL) and a soluble interleukin-2 receptor level of 594 U/mL (reference range, 192–530 U/mL).

**Figure 1.**
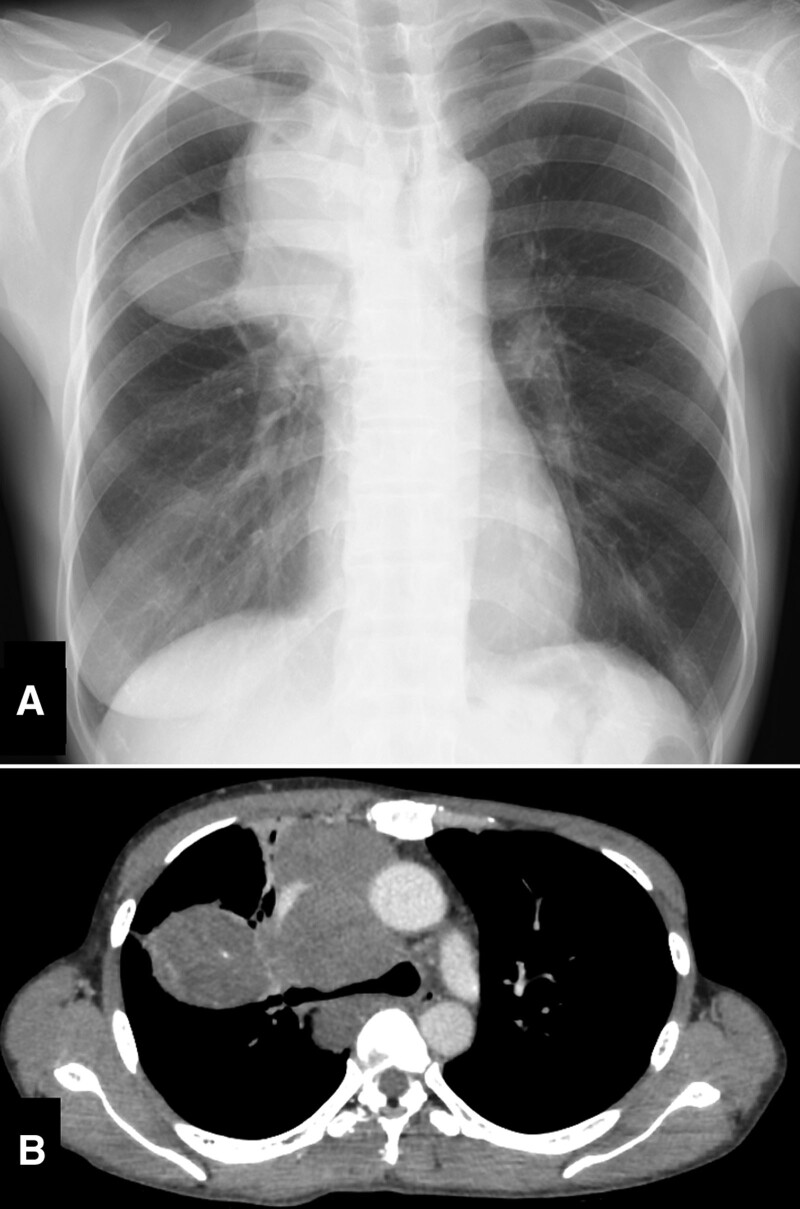
Pretreatment chest X-ray and contrast enhanced computed tomography of the tumor. (A) The chest X-ray showed a large mass in the right upper lobe without calcification. (B) The whole-body contrast enhanced (CE) computed tomography (CT) showed a large soft tissue mass 112 mm × 98 mm × 88 mm in size arising from the anterior mediastinum, expanding to the right lobe.

Chest radiography revealed a large mass in the right upper lobe without calcification (Fig. 1A). Whole-body contrast-enhanced CT showed a large soft tissue mass (112 mm × 98 mm × 88 mm) arising from the anterior mediastinum and expanding to the right lobe (Fig. 1B). No other masses were detected. Positron emission tomography-CT with fluorodeoxyglucose showed that the maximum standardized uptake value (SUV_max_) of the lesion was 11.3, and there were no other high fluorodeoxyglucose uptake sites. Magnetic resonance imaging of the head revealed no brain metastases.

**Figure 2.**
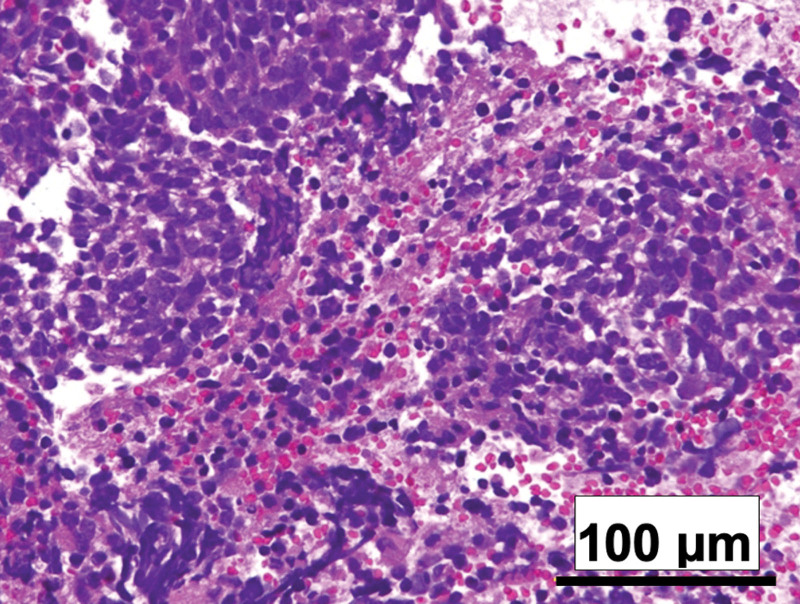
Histological examination of the biopsy specimen. The hematoxylin and eosin (HE) images showed the proliferation of small round cell tumor presenting naked nuclei with oval-, diamond-, or short spindle-shaped nuclei.

To confirm the pathological diagnosis, a second CT-guided biopsy was performed. Histopathological examination with hematoxylin and eosin staining indicated the presence of small round cell tumors. These tumors exhibited characteristics such as naked nuclei with oval-, diamond-, or short spindle-shaped nuclei (Fig. 2). Immunohistochemical analysis revealed that the tumor cells were positive for CD99/ MIC2, NSE, AE1/ AE3, and FLI-1. The cells stained negative for CD3, CD79a, TTF-1, chromogranin, and MyoD1. Considering these results, Ewing sarcoma was suspected, but a specific fusion gene was not detected owing to poor quality specimens.

**Figure 3.**
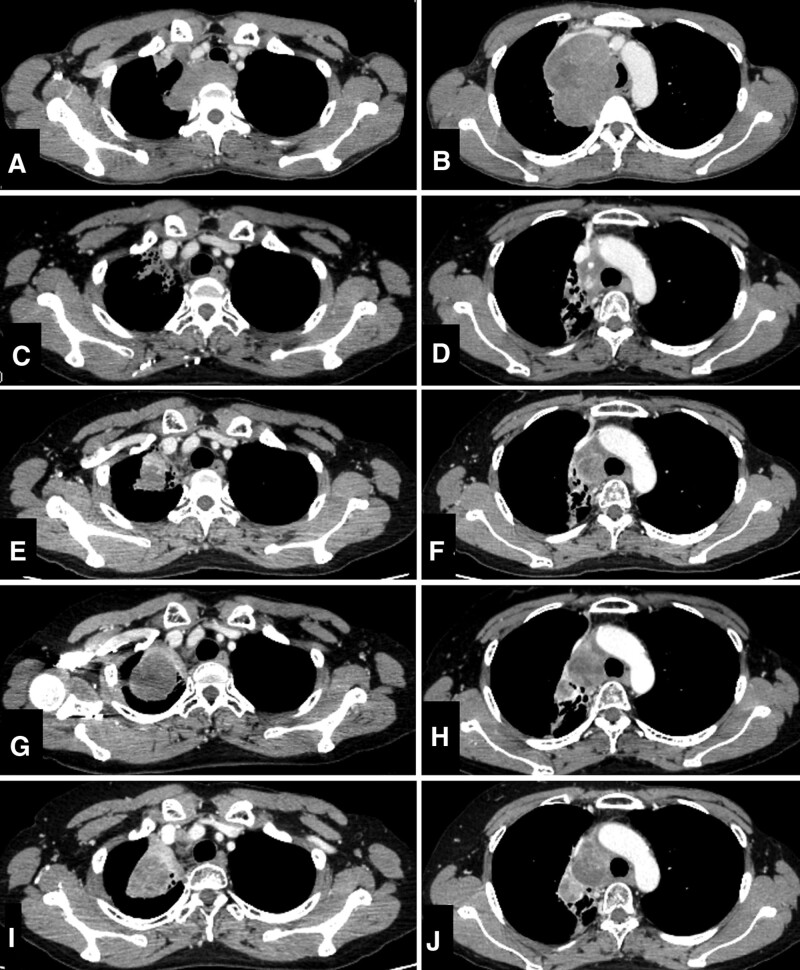
Contrast enhanced computed tomography of the tumor from pretreatment to third-line cytotoxic chemotherapy. When comparing the tumor images before chemotherapy (A, B) with those after 5 cycles of VDC/IE and radiotherapy (C, D), a significant reduction in tumor size was observed. However, following 2 cycles of VDC/IE, the tumor began to exhibit growth (E, F). Subsequently, after undergoing 2 cycles of topotecan and cyclophosphamide combination chemotherapy, the tumor growth persisted (G, H). The initiation of third-line chemotherapy consisting of 3 cycles of irinotecan and temozolomide led to stable disease, as indicated by the CT scans (I, J). CT = computed tomography, VDC/IE = Vincristine, doxorubicin, and cyclophosphamide, alternating with ifosfamide and etoposide.

As the breathing difficulty worsened and respiratory obstruction occurred, vincristine, doxorubicin, and cyclophosphamide alternating with ifosfamide and etoposide (VDC/IE) chemotherapy combined with radiotherapy, was initiated. After 5 cycles of VDC/IE and radiotherapy (54 Gy in 30 fractions), the tumor shrank markedly compared to the pretreatment tumor (Fig. 3A–D). Unfortunately, 2 months after the completion of radiotherapy, she developed radiation pneumonitis when 2 further cycles of VDC/IE chemotherapy had been completed. As the patient was treated for pneumonitis with oral prednisolone, the chemotherapy was suspended for a month. Considering the accumulation of doxorubicin, the 2 following cycles of chemotherapy were carried out with VC/IE excluding doxorubicin. Nevertheless, the tumor began to grow (Fig. 3E and F). Next, a combination of topotecan and cyclophosphamide was administered as second-line chemotherapy, but following this 2-cycle chemotherapy, the tumor continued to grow (Fig. 3G and H). Therefore, a combination of irinotecan and temozolomide (IT) was initiated as third-line chemotherapy. After 3 cycles of IT chemotherapy, CT showed stable disease (Fig. 3I and J). During IT chemotherapy, a transbronchial biopsy was performed, and the specimen was analyzed using next-generation sequencing, and *PHF20-NTRK1* fusion gene was detected. Following this, the tumor was finally diagnosed as *NTRK* fusion-positive sarcoma.

**Figure 4.**
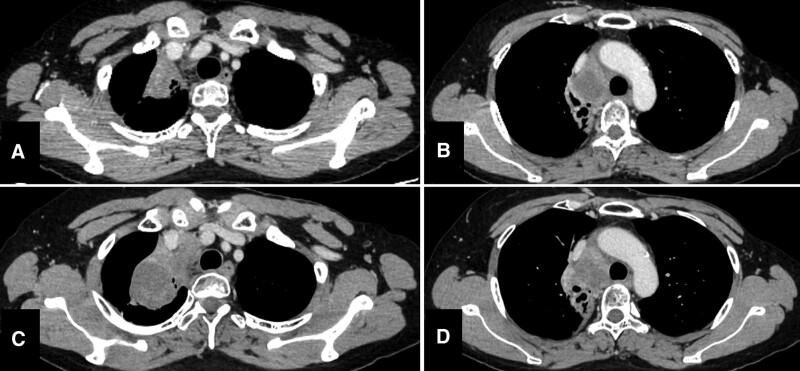
Contrast enhanced computed tomography of the tumor during entrectinib therapy. After 1 mo of entrectinib at 600 mg/day had been administered, CT showed stable disease (A, B). However, the tumor started to grow (C, D) after 5 mo of medication. CT = computed tomography.

After the diagnosis, the patient was administered entrectinib 600 mg/day, which was the only approved TRK inhibitor in Japan at that time. However, after 2 days of entrectinib at 600 mg/day had been administered, her neutrophils reduced to 730/μL. Consequently, she undertook a 20-day rest from entrectinib because her neutrophil count had not recovered. She restarted entrectinib at 400 mg/day for a week, and the dose increased to 600 mg/day. At 1 month of treatment after restarting entrectinib, the tumor diameter decreased by approximately 24% and was evaluated as stable disease according to the Revised Response Evaluation Criteria in Solid Tumors guideline version 1.1^[1]^ (Fig. 4A and B). At the same time, her neutrophils reduced to 870/μL; therefore, her dose of entrectinib was decreased to 400 mg/day. After 2 months of using 400 mg/day entrectinib, her neutrophils recovered to over 1000/μL, and the dose of entrectinib was increased to 600 mg/day again. Despite this, the tumor started to grow (Fig. 4C and D) after 5 months, and entrectinib was discontinued. Treatment-related adverse events were assessed according to the Common Terminology Criteria for Adverse Events version 5.0; entrectinib elicited grade 4 neutropenia, grade 3 leukopenia, grade 2 increased alanine aminotransferase, increased serum creatinine, increased aspartate aminotransferase, dysgeusia and grade 1 anemia, increased alkaline phosphatase, vertigo, and dizziness.

**Figure 5.**
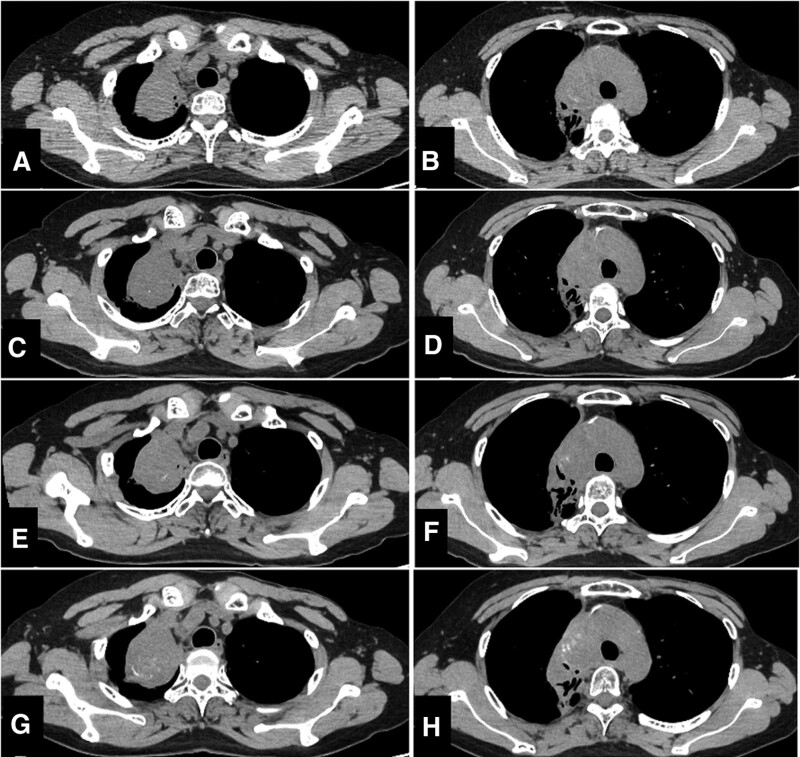
Computed tomography of the tumor during larotrectinib therapy. Larotrectinib treatment began at 200 mg orally daily; no dose reduction has been needed. The CT showed stable disease at 2 mo (A, B), 5 mo (C, D), 11 mo (E, F), and 21 mo (G, H) from commencement of larotrectinib treatment. CT = computed tomography.

Next, after 1 cycle of IT treatment, another TRK inhibitor, larotrectinib, which was the second TRK inhibitor approved in Japan, was administered when it became available in our hospital. Larotrectinib treatment was started at a daily dose of 200 mg orally, and no dose reduction was required. For more than 21 months after starting larotrectinib, she exhibited stable disease (Fig. 5A–H). Treatment-related adverse events, assessed according to common terminology criteria for adverse events version 5.0, were grade 1 leukopenia, anemia, increased alanine aminotransferase, increased aspartate aminotransferase, and grade 2 increased serum creatinine. She was also infected with COVID-19 after 8 months of treatment with larotrectinib and undertook a 22-day rest from larotrectinib. Altogether, she had been taking larotrectinib for more than 21 months and had maintained disease control at regular visits.

## 3. Discussion

*NTRK* fusion-positive tumors have been detected in a tissue-agnostic manner. The *NTRK* fusion detection rate differed significantly according to the original tissue. Solid tumors, such as sarcoma, colorectal cancer, and non-small cell carcinoma, that are positive for *NTRK* fusion result in at most about 0.68%.^[2]^ In contrast, more than 70% of infantile fibrosarcomas^[3,4]^ and more than 80% of mammary analog secretory carcinomas of the salivary gland are positive for *NTRK* fusion.^[5,6]^ When treated with standard therapies other than targeted TRK inhibitors, The prognosis of *NTRK* fusion-positive tumors is reportedly poorer than that of *NTRK* fusion-negative tumors derived from the same tissue type.^[7]^ TRK inhibitors are remarkably effective for such *NTRK* fusion-positive tumors. Indeed, the median duration of response and median progression-free survival of larotrectinib are reported as 35.2 and 28.3 months, respectively,^[8]^ in the same way those of entrectinib are reported as 20.0 and 13.8 months, respectively.^[9]^ Unfortunately, most *NTRK* fusion-positive tumors will eventually become resistant to these 2 novel TRK inhibitors. Resistance involves on-target resistance, involving a substitution in the solvent front, xDFG, or gatekeeper residue.^[10,11]^ As the second-generation anaplastic lymphoma kinase (ALK) inhibitor against ALK fusion-positive tumors is effective for on-target resistance to the first-generation ALK inhibitor,^[12]^ the second-generation TRK inhibitor selitrectinib^[13]^ or repotrectinib^[14]^ have been developed together with the first-generation TRK inhibitor, and their phase I/II trials are currently being conducted. However, only first-generation TRK inhibitors have currently been approved and are available in clinical practice.

It is not known if a first-generation TRK inhibitor, larotrectinib or entrectinib, is used and drug resistance emerges and whether the other first-generation TRK inhibitors would still be effective.

To the best of our knowledge, there is only 1 case in which 2 kinds of first-generation TRK inhibitors are administered sequentially; in a 34-year-old female with salivary acinic cell carcinoma with *ETV6-NTRK3* fusion gene arising from the left parotid.^[15]^ At first, she was diagnosed with salivary acinic cell carcinoma; therefore, first-to-third-line cytotoxic chemotherapy was administered.^[15]^ When *ETV6-NTRK3* fusion gene was detected, crizotinib, a multitarget kinase inhibitor, treatment was started and continued for 18 weeks until the progression of the disease.^[15]^ Subsequently, she began to take entrectinib,^[15]^ and after disease progression during 7 months of entrectinib treatment, larotrectinib was administered in the NAVIGATE study.^[16]^ Disease progression occurred within 1 month of larotrectinib treatment.^[16]^ Tumor sequencing of this patient conducted before beginning Larotrectinib revealed the solvent front mutation *NTRK3* G623R.^[15]^ *NTRK3* G623R mutation is known as a mutation that might arise in response to larotrectinib or entrectinib treatment.^[15,17]^ This mutation seems a likely candidate to have caused disease progression soon after the sequential TRK inhibitor treatment.^[15,16]^

By contrast, in our case, although entrectinib was administered as the first TRK multikinase inhibitor and her tumor became resistant to entrectinib and progressed, subsequent administration of larotrectinib, a highly selective TRK inhibitor, maintained stable disease for a long time. Therefore, resistance to entrectinib in the present patient might have occurred as off-target resistance or any site other than on-target resistance in the solvent front, xDFG, and gatekeeper residue. Consequently, the *NTRK* fusion gene in our patient was probably still a central oncogenic driver, and the conformation of the protein was conducive to larotrectinib interacting with the ATP binding site at the time when larotrectinib was administered.

One limitation of this case report is that DNA sequencing was not conducted again due to the lack of provision for second DNA sequencing in the Japanese health insurance system. Thus, while resistance to entrectinib emerged, the nature of the mutation remained unclear.

Pointedly, no country has yet approved the second-generation TRK inhibitors, and most countries offer the provision of DNA sequencing only once throughout such cases at the moment. For disease progression with 1 first-generation TRK inhibitor, it is possible that another first-generation TRK inhibitor can still be effective, as in our patient. Hence, another TRK inhibitor should be tried in cases where an *NTRK* fusion-positive tumor becomes resistant to a given single TRK inhibitor.

## 4. Conclusion

In this case report, we present a patient with *NTRK* fusion-positive sarcoma arising from the anterior mediastinum who has maintained disease control for more than 21 months and commenced larotrectinib only after the emergence of resistance to entrectinib. Thus, there are cases in which 2 approved first-generation TRK inhibitors can be used sequentially with clinical benefit.

## Acknowledgments

We would like to thank Editage (www.editage.com) for English language editing by the native English editors.

## Author contributions

**Conceptualization:** Yuta Kubota, Hiroshi Tsumura, Nobuhiro Kaku, Kazuhiro Tanaka

**Data curation:** Yuta Kubota, Masanori Kawano, Tatsuya Iwasaki, Ichiro Itonaga, Kazuhiro Tanaka

**Writing – original draft:** Yuta Kubota, Masanori Kawano, Tatsuya Iwasaki, Kazuhiro Tanaka

**Writing – review & editing:** Ichiro Itonaga, Hiroshi Tsumura, Nobuhiro Kaku, Kazuhiro Tanaka
